# Hypoxia/Reoxygenation-Induced Mitochondrial Reverse Electron Transfer: A Targetable Mechanism to Enhance Radiosensitivity in Non-Small Cell Lung Cancer

**DOI:** 10.3390/antiox15060697

**Published:** 2026-05-31

**Authors:** Cuilan Hu, Zheng Shi, Yanyu Bao, Nannan He, Xiongxiong Liu, Dan Xu, Qiang Li, Xingting Bao, Chao Sun

**Affiliations:** 1 Institute of Modern Physics, Chinese Academy of Sciences, Lanzhou 730000, China; hucuilan@impcas.ac.cn (C.H.); baoyanyu@impcas.ac.cn (Y.B.); lxx002@impcas.ac.cn (X.L.); xudan@impcas.ac.cn (D.X.); liqiang@impcas.ac.cn (Q.L.); 2 University of Chinese Academy of Sciences, Beijing 100190, China; 3 State Key Laboratory of Heavy Ion Science and Technology, Lanzhou 730000, China; 4 School of Biopharmaceutical and Engineering, Lanzhou Jiaotong University, Lanzhou 730070, China; shizheng@lzjtu.edu.cn; 5 Department of Critical Care Medicine, Lanzhou University Second Hospital, Lanzhou 730000, China; ldyy_henn@lzu.edu.cn; 6 Medical College, Northwest Minzu University, Lanzhou 730000, China

**Keywords:** mitochondrial ROS, reverse electron transfer, oxidative stress, radiosensitization, succinate

## Abstract

Hypoxia-induced radioresistance remains a major obstacle in non-small cell lung cancer (NSCLC) radiotherapy. This study investigates whether artificially activating mitochondrial reverse electron transfer (RET) can enhance radiosensitivity in NSCLC by triggering oxidative stress. An in vitro hypoxia/reoxygenation (H/R) model was established in A549 cells to assess reactive oxygen species (ROS) levels, mitochondrial function, and metabolic alterations using fluorescence probes, flow cytometry, confocal microscopy, and targeted metabolomics. Mitochondrial complex inhibitors and dimethyl succinate (DM-S) were employed to validate the RET mechanism, and radiosensitivity was evaluated through clonogenic survival, apoptosis assays, and γ-H2AX staining. In vivo, A549 tumor-bearing mice received high oxygen (95% O_2_) combined with DM-S and localized irradiation (4 Gy); tumor growth, histopathology, and immunohistochemistry were examined. H/R triggered substantial mitochondrial ROS production via complex I-mediated RET, dependent on a high mitochondrial membrane potential and electron transport chain imbalance, with succinate accumulation serving as a key metabolic switch. Exogenous DM-S exacerbated H/R-induced oxidative damage, DNA fragmentation (8-OHdG elevation, mtDNA integrity loss), and mitochondrial network disruption. H/R combined with DM-S significantly enhanced in vitro radiosensitivity, reducing clonogenic survival and increasing apoptosis to 53.4% ± 1.9% versus 10.3% ± 1.2% with irradiation alone. In vivo, the combination therapy markedly suppressed tumor growth, induced apoptosis and oxidative lipid damage (4-HNE), alleviated hypoxia (reduced HIF-1α), and showed no overt toxicity. These findings demonstrate that activating mitochondrial RET effectively enhances radiosensitivity in NSCLC. Succinate metabolism is a critical therapeutic target, and combining high oxygen with a succinate analog represents a promising radiosensitization strategy for hypoxic tumors.

## 1. Introduction

Non-small cell lung cancer (NSCLC) is one of the leading causes of cancer-related morbidity and mortality worldwide [1,2]. Despite advancements in treatment strategies, managing locally advanced and metastatic NSCLC remains a significant challenge [3,4,5]. In particular, the differential response of tumors to radiation therapy poses a major hurdle, with treatment failure commonly linked to the enhanced radioresistance of tumors [6,7,8]. Radiation resistance is influenced by a variety of factors, including the tumor hypoxic microenvironment [6], mutations in driver genes [9], and the intrinsic heterogeneity and evolution of the tumor [10].

Hypoxia is a hallmark feature of the tumor microenvironment, particularly evident in NSCLC [6]. Rapid tumor proliferation leads to vascular dysfunction, resulting in regions with oxygen tension lower than physiological levels, thereby creating a hypoxic microenvironment [11,12]. This state not only promotes tumor cell invasion, metastasis, and resistance to therapy, but also enhances DNA repair capabilities in tumor cells via the activation of hypoxia-inducible factors (HIFs) and other signaling pathways, thereby contributing to radiation resistance [13]. Tumor hypoxia can be classified into two types based on its underlying mechanisms: chronic hypoxia (diffusion-limited hypoxia) and acute hypoxia (perfusion-limited hypoxia) [14]. These two types together contribute to the complex and highly heterogeneous oxygenation state of solid tumors, which complicates therapeutic approaches [15]. Moreover, tumor hypoxia promotes the formation of cancer stem cells, which are key players in tumor metastasis, recurrence, and enhanced radiation resistance [16,17]. Cancer stem cells enhance the radiation resistance of NSCLC by activating the IGF1Rβ/PI3K/Akt signaling pathway [18].

Radiation therapy kills tumor cells by inducing lethal DNA damage either directly through ionizing radiation or indirectly through the generation of reactive oxygen species (ROS) via the radiolysis of water [19]. However, the role of ROS in cancer cells is multifaceted [20]. On one hand, moderate ROS levels protect mitochondria, and tumor cells accelerate ROS conversion to tolerate hypoxia, thus meeting the demands of rapid tumor growth [21]. Previous studies have shown that under hypoxic conditions, ROS production is suppressed, and hypoxia adaptation upregulates endogenous antioxidant systems (e.g., glutathione) to neutralize ROS [22], while regulating gene expression involved in DNA damage repair pathways [23]. This systemic enhancement of repair mechanisms increases cell survival and leads to the development of radiation resistance in tumor cells [23]. According to the widely accepted “oxygen fixation” hypothesis, the lack of oxygen prevents ROS-induced DNA damage from being “fixed” into lethal, irreparable double-strand breaks, thus reducing radiation sensitivity [17,24].

On the other hand, excessive ROS production can overcome the hypoxia-induced radiation resistance of cancer cells [25]. At high levels, ROS can induce oxidative stress, damaging DNA, proteins, and lipids, and activating cell death pathways [26,27]. Mitochondrial DNA is particularly vulnerable to oxidative damage due to its lack of histone protection and limited repair capabilities [28]. Damage to mitochondrial DNA further impairs electron transport chain function, leading to additional ROS leakage and creating a vicious cycle of “ROS-mitochondrial damage” [29]. This persistent mitochondrial dysfunction, particularly the loss of membrane potential and the aberrant opening of the mitochondrial permeability transition pore (mPTP), is a key event in initiating apoptotic and other cell death programs [30]. Numerous studies have shown that cells with impaired mitochondrial function (such as ρ0 cells) exhibit heightened sensitivity to radiation, with more severe nuclear DNA damage [31].

Previous studies have demonstrated that increasing arterial oxygen tension through the administration of high-oxygen gas mixtures can effectively enhance tumor tissue oxygenation, thereby improving the efficacy of radiation therapy [32]. However, these studies also noted that some tumor types remain poorly oxygenated even under high-oxygen conditions and require hyperbaric oxygen therapy for optimal therapeutic effects [33]. In recent years, alternative oxygenation strategies have garnered attention. For instance, inhibiting mitochondrial oxidative phosphorylation (OXPHOS) to reduce the oxygen consumption of tumor cells can increase the availability of oxygen within the tumor, thereby enhancing radiosensitivity [34]. Thus, enhancing tumor oxygenation, whether through high-oxygen interventions, vascular modulation, or optimizing oxygen delivery systems, has been considered a promising direction for improving tumor radiosensitivity [35,36]. However, the dynamic nature of tumor oxygenation levels and the complex regulatory network between the tumor microenvironment and oxygenation present significant challenges for developing oxygenation-based therapies.

Mitochondrial reverse electron transfer (RET) refers to the phenomenon in which electrons flow in the opposite direction across the mitochondrial inner membrane when the membrane potential is elevated, and the coenzyme Q (CoQ) pool is in a reduced state [37]. RET results in the production of large amounts of ROS, particularly superoxide (O_2_^•−^) [38]. Compared to the conventional forward electron transfer (FET), RET generates significantly more ROS, which are believed to play a crucial role in various cellular processes such as signaling, aging, and stress responses [39,40]. Notably, in ischemic conditions, the accumulation of metabolic products like succinate has been shown to induce RET, leading to a dramatic increase in ROS production and causing tissue damage [41]. While RET has been extensively studied in ischemia–reperfusion injury, its occurrence and potential therapeutic exploitation in the tumor context, particularly in hypoxic regions undergoing intermittent reoxygenation, remain largely unexplored.

Given the prevalence of the hypoxic tumor microenvironment, inducing RET in hypoxic tumor regions presents a promising strategy to enhance the radiosensitivity of hypoxic tumors. Accordingly, this study proposes a promising approach based on the artificial activation of mitochondrial RET to disrupt tumor cells from within, thereby enhancing their radiosensitivity. The specific hypothesis is as follows: intervention with high oxygen can improve the oxygenation status of hypoxic tumors and elevate oxygen supply, thereby facilitating the occurrence of mitochondrial RET and triggering a burst of ROS generation. This process not only induces cascading damage to mitochondrial and nuclear DNA but also severely disrupts cellular redox homeostasis, ultimately leading to a marked enhancement of radiosensitivity in hypoxic tumor regions.

## 2. Materials and Methods

### 2.1. Cell Culture

The human non-small cell lung cancer cell line A549 was obtained from Cos9X Bio (Suzhou, China). Cells were cultured in RPMI-1640 medium (Melun Bio, Dalian, China) supplemented with 10% fetal bovine serum (ExCell Bio, Suzhou, China). They were maintained in a humidified incubator at 37 °C with 5%CO_2_ and subcultured every 2–3 days using trypsin containing 0.25% EDTA (Melun Bio, Dalian, China). Phosphate-buffered saline (PBS) used for cell passage was purchased from Servicebio (Wuhan, China). Throughout the experiments, a consistent subculture regimen was followed to ensure that all samples were derived from the same parental line. For cryopreservation, cells were suspended in a mixture of 90% fetal bovine serum and 10% DMSO, stored overnight at −80 °C, and then transferred to liquid nitrogen for long-term storage. All experiments were performed using mycoplasma-free cells.

### 2.2. Mitochondrial DNA-Deleted (ρ0) Cells

A549 cells were cultured in the presence of 200 ng/mL ethidium bromide, 100 μg/mL pyruvate and 50 μg/mL uridine for up to 60 days [42]. ρ0 cells lack mitochondrial respiration due to loss of critical ETC proteins encoded by mitochondrial DNA. Depletion of mtDNA was confirmed by PCR amplification of cytochrome C oxidase subunit II (COX II). The sequence information of the primers was also listed in Table 1.

### 2.3. Detection of Intracellular Superoxide O_2_^•−^ by Electron Paramagnetic Resonance (EPR)

Intracellular superoxide levels in A549 and A549 ρ0 cells were detected by EPR using the spin trap DMPO (5,5-dimethyl-1-pyrroline N-oxide). After treatment, cells were collected and resuspended in Krebs-HEPES buffer containing 50 mM DMPO. The suspension was immediately transferred to a quartz capillary and placed into an EPR spectrometer (EPR200-Plus, CIQTEK, Hefei, China). EPR spectra were recorded at room temperature with the following parameters: center field, 3480 G; scan width, 100 G; microwave frequency, 9.78 GHz; microwave power, 10 mW; modulation amplitude, 1 G. Superoxide levels were quantified by measuring the peak amplitude of the characteristic DMPO-OOH adduct signal [43].

### 2.4. X-Ray Irradiation

X-ray irradiation was performed on both cells and nude mice using an X-ray irradiator (X-Rad 225, PXI Inc., Menifee, CA, USA) at the Institute of Modern Physics, Chinese Academy of Sciences, with a dose rate of approximately 1 Gy/min.

### 2.5. Detection of Intracellular ROS

The total intracellular ROS level was detected using the fluorescent probe 2′,7′-dichlorodihydrofluorescein diacetate (DCFH-DA). After treatment, cells were collected and seeded at an appropriate density into 96-well black-walled plates with clear bottoms. Following cell attachment, the culture medium was replaced with serum-free medium containing 10 μmol/L DCFH-DA, and the cells were incubated at 37 °C in the dark for 20 min. After incubation, the cells were gently washed twice with pre-warmed PBS to remove any unincorporated probe. Subsequently, 100 μL of PBS was added to each well, and fluorescence intensity was immediately measured using a microplate reader at an excitation wavelength of 485 nm and an emission wavelength of 535 nm. Cell-free blanks and unstained cell controls were included to account for background fluorescence. Each experimental group was assayed in at least five replicate wells, and the experiment was independently repeated three times. Fluorescence intensity data were corrected against blank values and used to compare relative ROS levels among groups.

### 2.6. Detection of Intracellular Superoxide by DHE

The intracellular oxidation product level was detected using the fluorescent probe dihydroethidium (DHE). Briefly, treated cells were collected, resuspended in pre-warmed PBS, and adjusted to an appropriate density. Cells were then incubated with DHE working solution (final concentration: 5 μmol/L) at 37 °C in the dark for 30 min. After incubation, cells were washed twice with ice-cold PBS to remove excess probe. Subsequently, cells were resuspended in an appropriate volume of PBS and immediately analyzed by flow cytometry. Unstained cells were used as a negative control. The excitation wavelength was set at 518 nm, and fluorescence emission was collected around 605 nm. At least 10,000 events were recorded per sample, and the experiment was independently repeated three times. The mean fluorescence intensity (MFI) of each group was calculated using flow cytometry analysis software to reflect the relative level of intracellular DHE oxidation products.

### 2.7. Detection of Mitochondrial Superoxide by MitoSOX

Mitochondrial-derived superoxide in cells was specifically detected using the MitoSOX Red fluorescent probe (Catalog No. M36008, ThermoFisher, Waltham, MA, USA). Cells were seeded into confocal dishes (BS-15-GJM, biosharp, Hefei, China) and allowed to adhere fully after treatment. The culture medium was then replaced with serum-free medium containing 5 μmol/L MitoSOX Red, followed by incubation at 37 °C in the dark for 20 min. After incubation, cells were washed twice with pre-warmed PBS to remove any unincorporated probe. Images were acquired using a Zeiss LSM-700 confocal microscope (Carl Zeiss, Jena, Germany) equipped with a Plan-Apo 40×/1.3 NA oil-immersion objective, with excitation/emission wavelengths set at 510/580 nm. Quantitative analysis of the images was performed using ImageJ 2 software (NIH, Bethesda, MD, USA), and the mean fluorescence intensity was used to reflect the relative level of mitochondrial superoxide. The experiment was independently repeated three times.

### 2.8. γ-H2AX Foci Detection

To evaluate the extent of DNA damage induced by different treatments and irradiation, γ-H2AX expression—a DNA damage marker corresponding to phosphorylated H2AX—was detected via immunofluorescence staining in A549 cells at 1 h after X-ray exposure. The experimental procedures were carried out according to the instructions of the γ-H2AX Immunofluorescence DNA Damage Assay Kit (Catalog No. C2035S; Beyotime Biotechnology, Shanghai, China). Confocal images were acquired using a Zeiss LSM-700 confocal microscope (Carl Zeiss, Jena, Germany) equipped with a Plan-Apo 40×/1.3 NA oil-immersion objective. Subsequent image processing was performed using Adobe Illustrator 2022 (Adobe Inc., San Jose, CA, USA).

### 2.9. Measurement of Mitochondrial Membrane Potential (ΔΨm)

Mitochondrial membrane potential (ΔΨm) was assessed using the fluorescent probe JC-10 (Beyotime Biotechnology, Shanghai, China). After the indicated treatments, a working solution of JC-10 was prepared at a concentration of 5 µg/mL in serum-free medium according to the manufacturer’s instructions. A549 cells were incubated with the JC-10 working solution at 37 °C in the dark for 30 min. Subsequently, cells were washed twice with ice-cold PBS to remove unbound dye. The stained cells were collected and analyzed on a CytoFLEX flow cytometer (Beckman Coulter, Brea, CA, USA). The fluorescence ratio of JC-10 monomers to aggregates was calculated using CytExpert 2.4 software to evaluate changes in mitochondrial membrane potential.

### 2.10. Apoptosis Assay

Apoptosis was detected and analyzed by flow cytometry using an Annexin V-FITC/PI double-staining assay. Briefly, cells were harvested after trypsin digestion following treatment and washed twice with ice-cold PBS. The cells were then resuspended in binding buffer and stained with Annexin V-FITC and propidium iodide (PI) by incubation in the dark at room temperature for 15 min. Stained cells were immediately analyzed on a flow cytometer (CytoFLEX, Beckman Brea, CA, USA).

### 2.11. Western Blot Analysis

Western blot analysis was performed to detect the expression levels of target proteins. Briefly, total protein was extracted from treated cells using RIPA lysis buffer containing protease inhibitors (Beyotime Biotechnology, catalog number P0013C, Shanghai, China), and protein concentration was quantified by the BCA method. Equal amounts of protein samples were separated by sodium dodecyl sulfate-polyacrylamide gel electrophoresis (SDS-PAGE) and transferred onto 0.45 μm PVDF membranes via wet transfer. After transfer, the membranes were blocked with rapid blocking buffer at room temperature for 10 min. Subsequently, the membranes were incubated with corresponding primary antibodies (see Table 2) at 4 °C for approximately 12 h. The next day, the membranes were washed three times with TBST and then incubated with horseradish peroxidase-conjugated secondary antibodies (dilution 1:5000) at room temperature for 1 h. After thorough washing with TBST, signals were developed and captured using an enhanced chemiluminescence substrate on an AI680 chemiluminescence imaging system. β-Actin was used as the loading control, and the grayscale values of target bands were analyzed with ImageJ 2 software to calculate the relative protein expression levels.

### 2.12. Quantification of Mitochondrial DNA Damage Using Long PCR

MtDNA damage was analyzed by quantitative long PCR performed on an Eppendorf Mastercycler PCR system (Eppendorf, Hamburg, Germany). Primer sequences are listed in Table 3. The reaction system and conditions for long PCR are shown in Table 4 and Table 5, respectively. Equal amounts of the large PCR product were separated on a 0.5% vertical agarose gel and electrophoresed in TBE buffer for 2 h, while the small PCR product was separated on a 1% agarose gel and electrophoresed in TAE buffer for 1 h. Gels were digitally imaged, and signals were quantified using a FluorChem FC2 system (Alpha Innotech Corporation, San Leandro, CA, USA). The extent of mtDNA damage was assessed by comparing the relative amplification efficiency of the large fragment (16.2 kb, mtDNA) to that of the small fragment (956 bp, mtND1), with the latter serving as an internal control for normalization. For conventional PCR amplification (956 bp), the reaction was performed using 2× Es Taq MasterMix (Dye) (CWBIO, Beijing, China; Cat# CW0690M). For long PCR (16 kb), the GoTaq^®^ Long PCR Master Mix (Promega, Madison, WI, USA; Cat# M4021), which contains a blend of hot-start recombinant Taq DNA polymerase and a proofreading DNA polymerase, was used according to the manufacturer’s instructions.

### 2.13. Targeted Metabolomics Analysis

Targeted absolute quantitation analysis of energy metabolism-related metabolites was performed by Biapps Biotech (Shanghai, China), utilizing an LC-MS/MS-based targeted metabolomics platform. Targeted metabolomics analysis of energy-related metabolites was performed using a Shimadzu Nexera X2 LC-30AD ultra-high-performance liquid chromatography system (Shimadzu, Kyoto, Japan) coupled with an AB SCIEX QTRAP 6500+ mass spectrometer (AB SCIEX, Framingham, MA, USA). Chromatographic separation was carried out on a Phenomenex Kinetex F5 column (2.6 μm, 3 × 100 mm) maintained at 40 °C. The mobile phase consisted of (A) 10 mM ammonium acetate in water and (B) pure acetonitrile. The flow rate was 200 μL/min, and the injection volume was 5 μL. The gradient elution program was as follows: 0–3 min, 0% B; 3–8 min, linear gradient from 0% to 95% B; 8–10 min, hold at 95% B; 10–11 min, linear gradient from 95% to 0% B; 11–12 min, hold at 0% B for column equilibration. The autosampler temperature was maintained at 4 °C. Mass spectrometry was conducted using an AB SCIEX QTRAP 6500+ (AB SCIEX, Framingham, MA, USA) mass spectrometer equipped with an electrospray ionization (ESI) source operating in both positive and negative ion modes. Multiple reaction monitoring (MRM) mode was employed for data acquisition. The ESI source parameters were set as follows: ion source temperature, 500 °C; Ion Source Gas1 (GAS1), 55; Ion Source Gas2 (GAS2), 60; Curtain Gas (CUR), 35. The Ion Spray Voltage Floating (ISVF) was set at +5500 V for positive mode and −4500 V for negative mode. Data acquisition and peak integration were performed using MultiQuant 3.0.2 software (AB SCIEX). Metabolites were identified by comparing retention times and MRM transitions with authentic standards. The absolute concentrations of metabolites were calculated based on standard curves constructed for each metabolite.

### 2.14. Ultrasound and Photoacoustic Imaging of Oxygen Saturation (SO_2_) in Tumor Xenografts

To form a tumor xenograft, athymic BALB/c nude mice (Beijing HFK Bioscience, Beijing, China) were subcutaneously injected at hind-limb with the A549 cell suspension. 14 days later, the ultrasound contrast agent was intravenously injected into mice, and the ultrasound and photoacoustic imaging of xenograft tumor was performed using the Vevo LAZR photoacoustic imaging system (VisualSonics, Toronto, ON, Canada). All images including B-Mode imaging for high-resolution anatomical images, DCEUS for functional tissue perfusion and PA imaging for SO_2_ were generated with the LZ250 transducer at 21 MHz [44].

### 2.15. STED Imaging of Mitochondria

A549 cells were stained with 200 nM PK Mito Orange (GenVivo, Inc., San Diego, CA, USA) in DMEM for 30 min, and washed thrice with the culture medium. STED imaging was performed with a Leica STELLARIS 8 STED system using a 100× oil-immersion objective (Leica, Wetzlar, Germany, N.A. 1.4). PK Mito Orange was excited at 561 nm, and a 775 nm pulsed laser was used for STED depletion [45].

### 2.16. Electrochemical Analysis of H_2_O_2_ Release from A549 Cells at the Single-Cell Level

Electrochemical detection was performed using a three-electrode system in a single-cell analyzer. The microelectrode probe was first modified: the Ag/AgCl reference electrode, platinum wire counter electrode, and the probe were immersed in a hydrochloric acid solution (pH 2.0) containing 1 mM FeCl_3_, 1 mM K_3_[Fe(CN)_6_], and 0.1 M KCl, and subjected to potentiostatic deposition at +0.4 V for 100 s. Subsequently, the modified probe was transferred to a hydrochloric acid solution (pH 2.0) containing 0.1 M KCl, and characterized by cyclic voltammetry in the range of −0.2 V to +0.6 V at a scan rate of 50 mV/s for two cycles. The appearance of a distinct redox peak near +0.2 V indicated successful modification. A549 adherent cells cultured in 35 mm dishes were washed three times with PBS, followed by the addition of 2 mL PBS, and then placed on the stage of the single-cell analyzer. The three-electrode system was immersed in the cell bath solution, and the tip of the microelectrode probe was precisely positioned adjacent to an individual cell using a micromanipulator. Amperometric i-t curve measurement was employed with an applied potential of +0.6 V for a continuous detection period of 2000 s. After the baseline stabilized, 2 μL of the stimulant DM-S was added at 200 s, and 2 μL of PBS was added at 800 s as a negative control. The oxidative current response generated by H_2_O_2_ released from the cells was recorded in real time.

### 2.17. Statistical Analysis

All experiments were performed at least three independent times. Data are presented as mean ± SEM. Normality of distribution was assessed using the Shapiro–Wilk test. For comparisons between two groups, the unpaired two-tailed Student’s *t*-test was used when data were normally distributed and variances were equal. For comparisons involving three or more groups, one-way analysis of variance (ANOVA) was performed, followed by Tukey’s post hoc test for multiple comparisons. * *p*-value < 0.05 was considered statistically significant. Quantification of γ-H2AX foci and Western blot results was performed using ImageJ 2 software (National Institutes of Health, Bethesda, MD, USA). Statistical analyses and graph generation were conducted with GraphPad Prism 9 (GraphPad Software, San Diego, CA, USA).

### 2.18. In Vivo Experiments

Female athymic nude mice (6 weeks old) were purchased from Cavens Laboratory Animal Co., Ltd. (Changzhou, China). Although A549 cells were originally derived from a male patient, this cell line is not hormone-dependent. It has been shown to lack oestrogen receptor expression by ligand binding assay and Northern blotting, and 17-β-oestradiol does not affect its proliferation [46]. Therefore, host sex is unlikely to influence tumor growth. Female mice were chosen to minimize fighting and stress-induced immune variability. A total of 20 mice were subcutaneously inoculated with 5 × 10^6^ A549 cells in the axillary region. Tumor volumes were monitored, and approximately two weeks later, the mice were randomly allocated into 4 groups (*n* = 5) with comparable mean baseline tumor volumes: Control, C + 4 Gy, High oxygen + 4 Gy, and High oxygen + DM-S + 4 Gy. Following irradiation, tumor growth was observed for three weeks. The mice were then euthanized, and tumor tissues were excised and fixed in 4% paraformaldehyde for subsequent analysis. All animal procedures strictly followed the animal experimentation regulations in China and were approved by the Animal Ethics Committee of the Institute of Modern Physics of the Chinese Academy of Science (No.2025(005), on 3 March 2025).

Mice were placed in an induction chamber connected to medical-grade 100% oxygen (outlet to a waste gas scavenger). Chamber oxygen concentration was continuously monitored (>95%) using an oxygen sensor. Mice received hyperoxia treatment for 15 min without isoflurane anesthesia. Immediately thereafter, isoflurane anesthesia was introduced, and each mouse was transferred onto a lead shield for tumor-directed X-ray irradiation. The interval between the end of hyperoxia and the start of irradiation was strictly kept within 2–3 min.

### 2.19. H/R Model Establishment

To model dynamic oxygenation changes in the tumor microenvironment, A549 cells were subjected to hypoxia/reoxygenation (H/R) using a Billups-Rothenberg modular incubator chamber placed in a standard cell culture incubator (37 °C, 5% CO_2_, >95% humidity). Hypoxia was achieved by flushing the chamber with a pre-mixed gas (0.5% O_2_, 5% CO_2_, balance N_2_) for 5 min, followed by a 12 h incubation. Oxygen concentration was continuously monitored using an electrochemical oxygen electrode (MOM5003, Shenzhen Coolrun Life Science Technology Co., Ltd., Shenzhen, China), which does not require calibration. Reoxygenation was performed by replacing the hypoxic medium with fresh pre-warmed medium pre-equilibrated with hyperoxic gas (95% O_2_, 5% CO_2_) for 1 h, then flushing the chamber with the same gas for 3 min, reaching 95% O_2_. Cells were then incubated for an additional 2 h unless otherwise indicated. Medium pH was stable (phenol red indicator, pH 7.2–7.4), and humidity was maintained >95% using an open sterile water dish. All experiments were repeated at least three times independently.

### 2.20. Measurement of Malondialdehyde (MDA) Content

The content of malondialdehyde (MDA) in cell samples was determined using an MDA assay kit (Solarbio, Beijing, China, catalog number BC0025). After collecting the treated cells, the procedures were performed strictly according to the manufacturer’s instructions. Following the reaction, absorbance was measured at the appropriate wavelength using a microplate reader, and MDA concentration was calculated based on the formula provided in the kit. MDA levels reflect the extent of lipid peroxidation within the cells.

### 2.21. Complex V (ATP Synthase) Activity Assay

Complex V (ATP synthase) activity was measured using a commercial assay kit (Complex V/ATP Synthase Activity Assay Kit, BC1445, Solarbio, Beijing, China) following the manufacturer’s protocol.

## 3. Results

### 3.1. Hypoxia/Reoxygenation (H/R) Induces Mitochondrial Oxidative Stress in Tumor Cells

**Figure 1:**
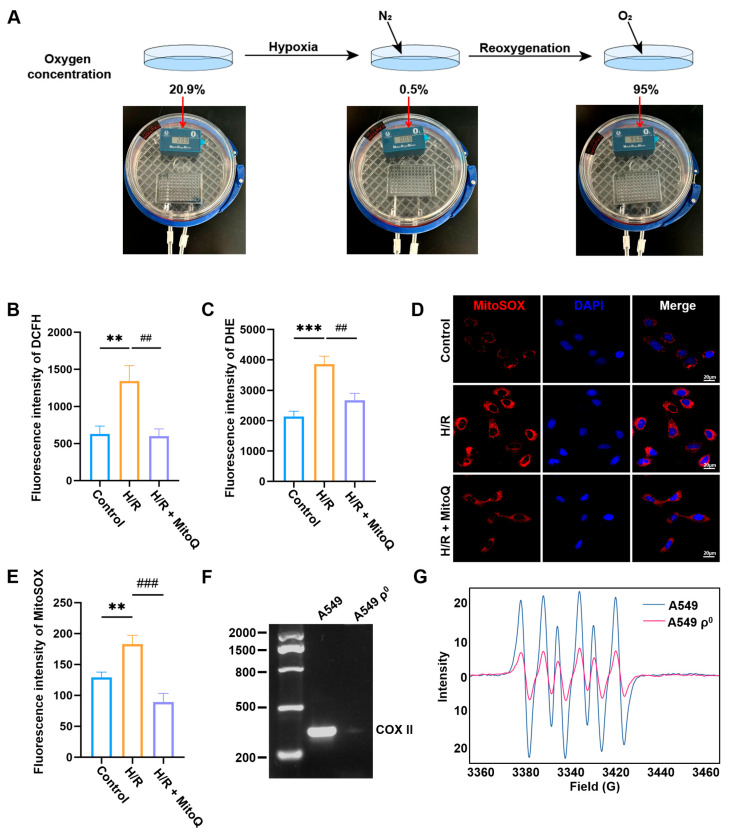
H/R Induces Mitochondrial Oxidative Stress in Tumor Cells. (**A**) Schematic of the experimental setup showing the transition of tumor cells from normoxic (20.9% O_2_) to hypoxic (0.5% O_2_) conditions followed by reoxygenation (95% O_2_) to simulate the tumor microenvironment’s shift in oxygen levels. (**B**) Detection of total ROS levels in tumor cells treated with H/R using the DCFH-DA probe and microplate reader (*n* = 3). (**C**) Flow cytometry analysis of DHE oxidation product levels using DHE staining (*n* = 3). (**D**,**E**) Confocal microscopy images and quantitative analysis of fluorescence intensity generated using the mitochondrial-targeted superoxide probe MitoSOX (*n* = 5). (**F**) Gel image confirming the absence of mtDNA in the A549ρ0 cell line used for further experiments. (**G**) EPR spectroscopy of A549 and A549ρ0 cells under H/R conditions (*n* = 3). All the data are presented as mean ± SEM from at least three independent experiments, and error bars represent SEM. Statistical significance between groups was analyzed using one-way ANOVA, followed by Tukey’s post hoc test for multiple comparisons. ** *p* < 0.01, *** *p* < 0.001 compared to normoxic control; ## *p* < 0.01, ### *p* < 0.001 compared to the H/R group.

To model the dynamic oxygenation changes in tumor hypoxic regions, A549 cells were subjected to hypoxia (0.5% O_2_, 12 h) followed by rapid reoxygenation (95% O_2_, 2 h), hereafter referred to as H/R treatment (Figure 1A). H/R significantly increased intracellular total ROS levels, as evidenced by a 2.13-fold elevation in DCFH-DA fluorescence compared to normoxic controls (Figure 1B). Similarly, DHE staining, which specifically detects DHE oxidation products, showed a marked 1.8-fold increase following H/R (Figure 1C).

To trace the cellular source of ROS, we employed the mitochondrial-targeted superoxide probe MitoSOX Red. Confocal microscopy revealed a dramatic 2.05-fold increase in mitochondrial superoxide signals in H/R-treated cells (Figure 1D,E), suggesting mitochondria as the primary origin. Pre-treatment with the mitochondria-specific antioxidant MitoQ (0.5 μM) markedly attenuated H/R-induced ROS elevation, reducing DCFH-DA fluorescence by 55.3% ± 1.1%, DHE fluorescence by 30.7% ± 1.4%, and MitoSOX fluorescence by 29.1% ± 6.5% (Figure 1B–E), further confirming the mitochondrial source.

We generated an A549ρ0 cell line lacking mtDNA (Figure 1F). EPR spectroscopy analysis revealed that under the same H/R conditions, the superoxide anion signal detected in A549ρ0 cells was significantly weaker than that in wild-type cells (Figure 1G). This result is consistent with a mitochondrial contribution to H/R-induced ROS, though we acknowledge that Rho-0 cells have extensive metabolic adaptations beyond electron transport chain loss. Together with the MitoSOX and MitoQ data (Figure 1D,E), these findings support the involvement of mitochondria as a primary source of ROS under H/R conditions.

Collectively, these results demonstrate that H/R treatment induces robust mitochondrial oxidative stress in A549 cells, establishing a foundation to investigate the underlying mechanisms of mitochondrial ROS production under dynamic oxygenation conditions.

### 3.2. H/R-Induced Mitochondrial ROS Are Generated via Complex I-Mediated Reverse Electron Transfer

**Figure 2:**
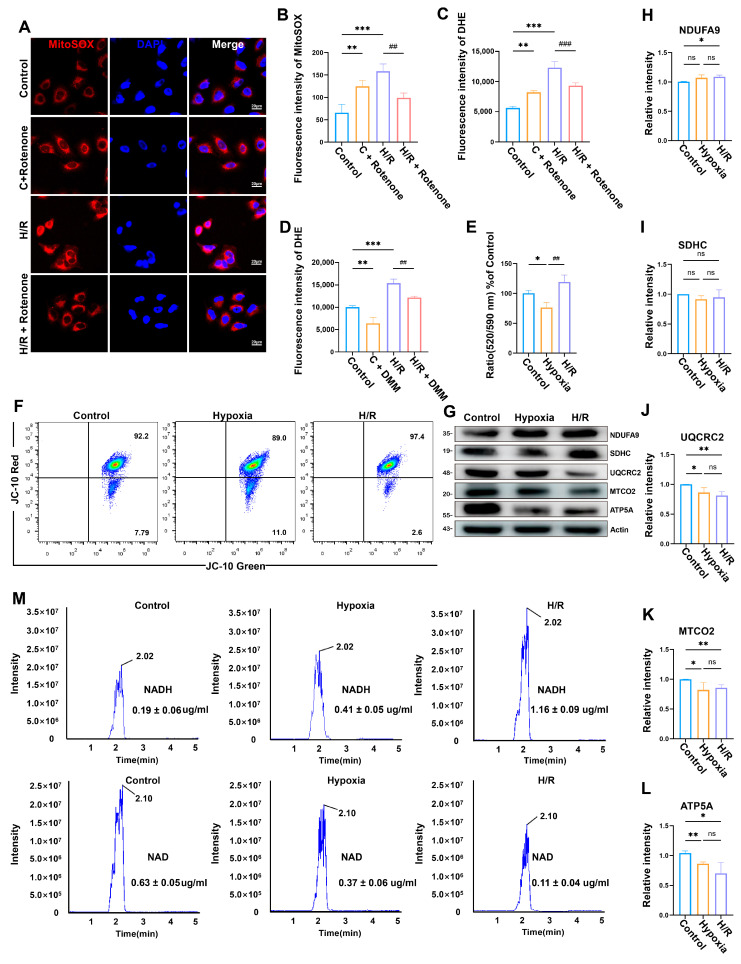
H/R-Induced Mitochondrial ROS Are Generated via Complex I-Mediated Reverse Electron Transfer. (**A**,**B**) Confocal microscopy images of mitochondrial ROS production following H/R and rotenone treatment, with quantitative fluorescence analysis (*n* = 5). (**C**) Quantitative analysis of DHE fluorescence intensity, reflecting DHE oxidation product levels. (**D**) Quantitative analysis of DHE fluorescence intensity with DMM treatment (*n* = 3). (**E**,**F**) ΔΨm was analyzed by flow cytometry using JC-10 staining, and the JC-10 aggregate/monomer ratio was quantitatively assessed in each group (*n* = 3). (**G**–**L**) Western blotting and quantitative analysis of key mitochondrial proteins (NDUFA9, SDHC, UQCRC2, MTCO2, ATP5A) (*n* = 3). (**M**) Energy metabolism analysis by mass spectrometry. Quantification of mitochondrial NADH and NAD levels in control, hypoxic, and H/R-treated A549 cells (*n* = 3). Data are presented as mean ± SEM. All the data are presented as mean ± SEM from at least three independent experiments, and error bars represent SEM. Statistical significance between groups was analyzed using one-way ANOVA, followed by Tukey’s post hoc test for multiple comparisons. “ns” represents no statistical difference, * *p* < 0.05, ** *p* < 0.01, *** *p* < 0.001 compared to the control group; ## *p* < 0.01, ### *p* < 0.001 between the indicated groups.

To identify the primary source of mitochondrial ROS production under H/R conditions, we used rotenone, a specific inhibitor of mitochondrial complex I. Under normoxic conditions, rotenone treatment (which blocks FET) increased DHE oxidation product levels as expected (Figure 2A–C), consistent with the classical mechanism by which electron accumulation at complex I enhances ROS generation. However, compared with the control group, rotenone treatment significantly suppressed ROS levels under H/R conditions (Figure 2A–C). The opposing effects of rotenone on ROS levels under normoxic versus H/R conditions strongly suggest that H/R-induced ROS do not originate from complex I-mediated FET, but are more likely driven by RET at this complex.

To assess whether RET occurs, we examined two key prerequisites for its initiation: a high ΔΨm and an imbalance in electron transport chain (ETC) activity. Measurement of ΔΨm (Figure 2E,F) showed that H/R treatment effectively reversed the hypoxia-induced decline in ΔΨm, restoring it to a high level, thereby providing the necessary proton motive force to drive electron flow from the reduced ubiquinone pool back to complex I. Further analysis of ETC complex activities (Figure 2G–L) revealed that after H/R, the protein expression levels of complexes I and II were maintained or even enhanced, whereas the protein expression levels of downstream complexes III, IV, and ATP synthase (complex V) were significantly reduced. This process was accompanied by a marked decrease in complex V (ATP synthase) enzymatic activity (Figure S1) and a reduction in overall cellular oxygen consumption rate (Figure S2). This imbalance, characterized by relatively dominant upstream complex activity, creates favorable conditions for RET.

Notably, although complex II itself is not a major direct source of ROS, its functional status can indirectly influence RET and ROS generation by modulating the electron flow toward complex I. To further investigate the role of complex II in this process, we used its specific inhibitor, dimethyl malonate (DMM). DMM treatment significantly attenuated H/R-induced ROS levels (Figure 2D). This result suggests that inhibition of complex II function under H/R conditions reduces the electron reflux driving RET, thereby diminishing ROS production.

Furthermore, we performed energy metabolism analysis by mass spectrometry, which revealed a significant accumulation of NADH and a marked decrease in NAD levels in mitochondria after H/R treatment (Figure 2M). This metabolic shift provides direct evidence that complex I operates in RET mode rather than the classical FET mode under H/R conditions.

Together, these data demonstrate that H/R induces mitochondrial ROS production through complex I-mediated RET, driven by high ΔΨm, ETC imbalance, and an increased NADH/NAD ratio.

### 3.3. Changes in Energy Metabolites During the RET Process: Succinate as a Potential Metabolic Switch

**Figure 3:**
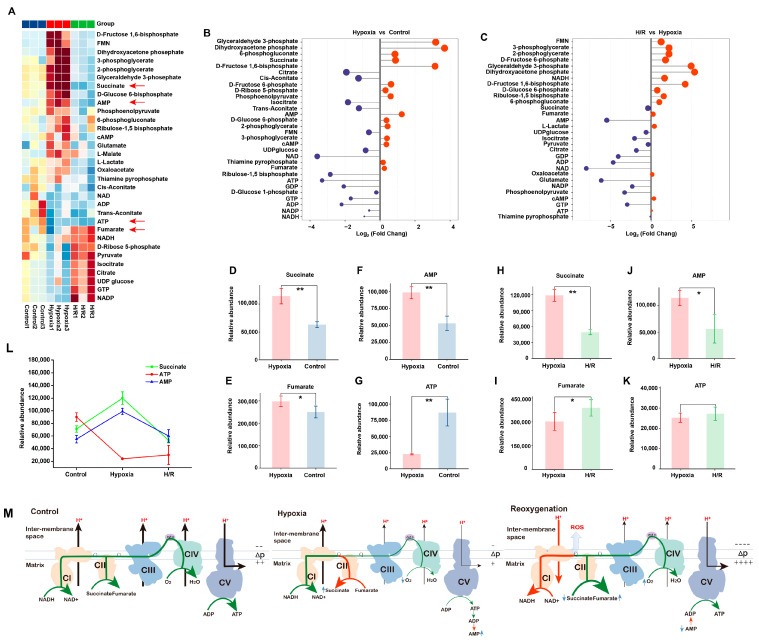
Changes in Energy Metabolites During the RET Process. (**A**) Heatmap of metabolic alterations (*n* = 3): The heatmap displays the differential expression of metabolites under different experimental conditions. Colors represent the relative abundance of each metabolite across the various groups. The red arrows indicate the key metabolites of interest. (**B**) Differential metabolite levels between hypoxia and control groups: Volcano plot showing metabolites that are significantly upregulated (orange) or downregulated (purple in hypoxia compared to the control group. (**C**) Differential metabolite levels between H/R and hypoxia groups. (**D**–**K**) Quantification of specific metabolites: Bar graphs show the relative abundance of metabolites including succinate, AMP, fumarate, and ATP, under different conditions (Hypoxia vs. Control, and H/R vs. Hypoxia). (**L**) Changes in the relative abundance of succinate, ATP, and AMP under different conditions. (**M**) Schematic diagram of RET metabolism. Green arrows indicate normal FET; red arrows indicate RET; “+/−” signs represent membrane potential. All the data are presented as mean ± SEM from at least three independent experiments, and error bars represent SEM. Statistical significance between groups was analyzed using an unpaired *t*-test. * *p* < 0.05, ** *p* < 0.01.

Building on the metabolic dynamics associated with the occurrence of RET, we further conducted metabolomic analysis (Figure 3A–C). The results showed that AMP levels significantly increased under hypoxic conditions, while they were rapidly consumed after H/R. ATP exhibited an opposite trend, decreasing significantly following hypoxia and recovering slightly after H/R without statistical significance (Figure 3F,G,J–L). These findings further confirm that during RET, the normal function of the mitochondrial respiratory chain is impaired, leading to abnormal cellular energy metabolism.

Additionally, the analysis uncovered a key metabolic shift: succinate markedly accumulated during hypoxia and was rapidly oxidized upon subsequent reoxygenation (Figure 3D,H,L). In contrast, fumarate significantly increased after H/R. This trend aligns with established ischemia–reperfusion injury models, where succinate accumulation is a core metabolic event driving RET [41]. Our study confirms that, in the tumor cell H/R model, succinate accumulation similarly provides the necessary reducing potential for RET, forming its metabolic foundation.

Under hypoxic conditions, complex II operates in reverse by reducing fumarate to succinate, acting as the terminal electron acceptor in place of oxygen. The reduced flux through the rest of the electron transport chain results in insufficient ATP generation, leading to an increase in AMP production from ADP (Figure 3M). Upon reoxygenation, complex II metabolism restores the accumulated succinate. At this point, the mitochondrial membrane becomes hyperpolarized, and the highly reduced state forces Complex I to operate in reverse, accompanied by the conversion of NAD to NADH (Figure 2M) and triggering substantial ROS production. AMP is then converted back to ADP, and ATP production gradually recovers. The flux through the electron transport chain and the citric acid cycle subsequently normalizes. Our metabolomic data implicate succinate accumulation as a key metabolic correlate of RET activation under H/R conditions, leading us to functionally validate its causal role in the subsequent experiments.

### 3.4. Exacerbation of H/R-Induced Oxidative Damage by Exogenous Dimethyl Succinate (DM-S)

**Figure 4:**
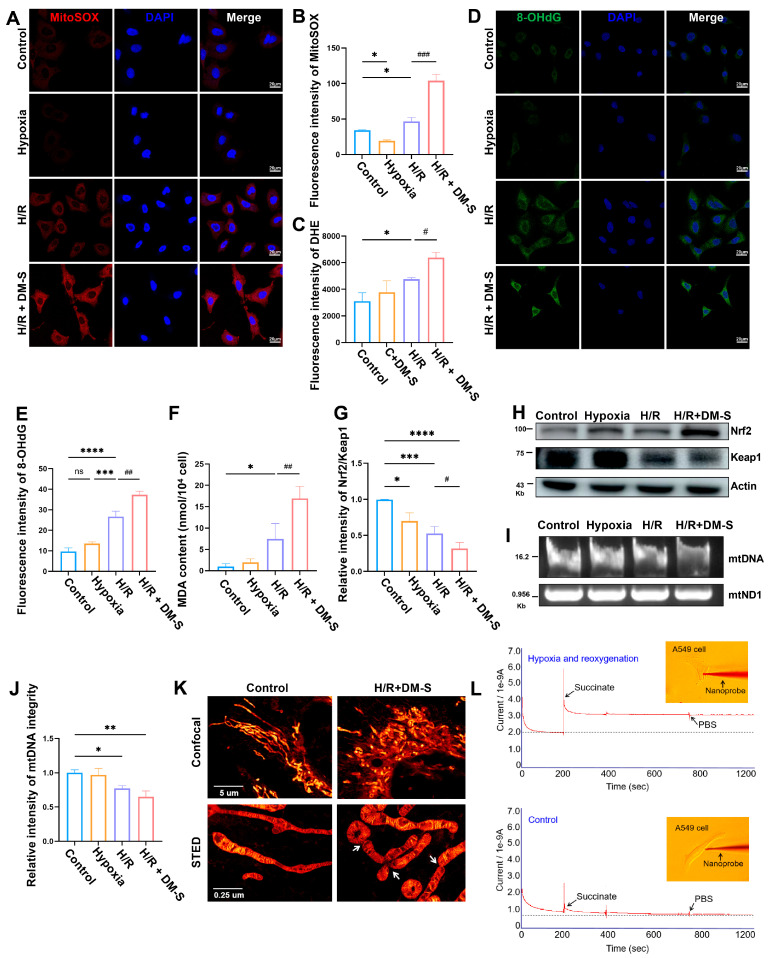
Exacerbation of H/R-induced oxidative damage by exogenous DM-S. (**A**,**B**) Representative confocal images and quantitative analysis of mitochondrial superoxide levels (MitoSOX Red) in A549 cells treated with H/R or H/R + DM-S (*n* = 5). (**C**) Quantitative analysis of DHE fluorescence intensity, reflecting DHE oxidation product levels (*n* = 3). (**D**,**E**) Representative images and quantitative analysis of oxidative DNA damage marker 8-hydroxy-2′-deoxyguanosine (8-OHdG) (*n* = 5). (**F**,**G**) Quantification of MDA levels, a marker of lipid peroxidation (*n* = 3). (**H**) Western blot analysis showing upregulation of Nrf2 and downregulation of its repressor Keap1 in the H/R + DM-S group (*n* = 3). (**I**,**J**) Long PCR was used to detect mtDNA integrity and quantitative analysis (*n* = 3). (**K**) Analysis of mitochondrial morphology showing pronounced fragmentation in the H/R + DM-S group (*n* = 3). The white arrows indicate the sites of mitochondrial fragmentation. (**L**) Real-time monitoring of intracellular H_2_O_2_ levels using a single-cell electrochemical platform (*n* = 3). The red line represents the real-time current signal of H_2_O_2_. Statistical Analysis: Data are presented as mean ± SEM. Error bars represent SEM, and statistical significance between groups was analyzed using one-way ANOVA, followed by Tukey’s post hoc test for multiple comparisons. “ns” represents no statistical difference, * *p* < 0.05, ** *p* < 0.01, *** *p* < 0.001, **** *p* < 0.0001 compared to the control group; # *p* < 0.05, ## *p* < 0.01, ### *p* < 0.001 compared to the H/R group.

To establish a causal role for succinate accumulation in H/R-induced RET and oxidative damage, we supplemented the H/R model with the membrane-permeable succinate analog DM-S. Exogenous DM-S significantly enhanced mitochondrial superoxide production, increasing MitoSOX Red fluorescence by 33.9% ± 8.5% compared to H/R alone (Figure 4A,B) and elevating intracellular superoxide levels by 1.2-fold (Figure 4C).

This exacerbated oxidative stress led to more severe cellular damage. 8-OHdG immunofluorescence, a marker of oxidative DNA damage, increased by 15.7% ± 6.9% in the DM-S + H/R group compared to H/R alone (Figure 4D,E). Long-range PCR analysis revealed that intact mtDNA was reduced by 35.0% ± 8.5% following DM-S + H/R treatment (vs. Control, Figure 4I,J), indicating significant mtDNA damage. Lipid peroxidation, assessed by MDA levels, was also significantly increased (Figure 4F,G).

Concurrently, the Nrf2/Keap1 antioxidant axis was disrupted, with upregulated Nrf2 and downregulated Keap1 expression (Figure 4G,H). Mitochondrial network analysis revealed pronounced fragmentation following DM-S + H/R treatment (Figure 4K). Real-time single-cell electrochemical monitoring further confirmed that succinate triggers a transient H_2_O_2_ burst specifically upon H/R, but not under normoxia (Figure 4L).

Collectively, these multi-layered findings demonstrate that succinate acts as a critical metabolic trigger driving RET and explosive oxidative stress under H/R conditions, establishing a key mechanistic link between dynamic oxygenation changes and mitochondria-dependent cellular injury.

### 3.5. H/R Combined with DM-S Synergistically Enhances Radiosensitivity In Vitro

**Figure 5:**
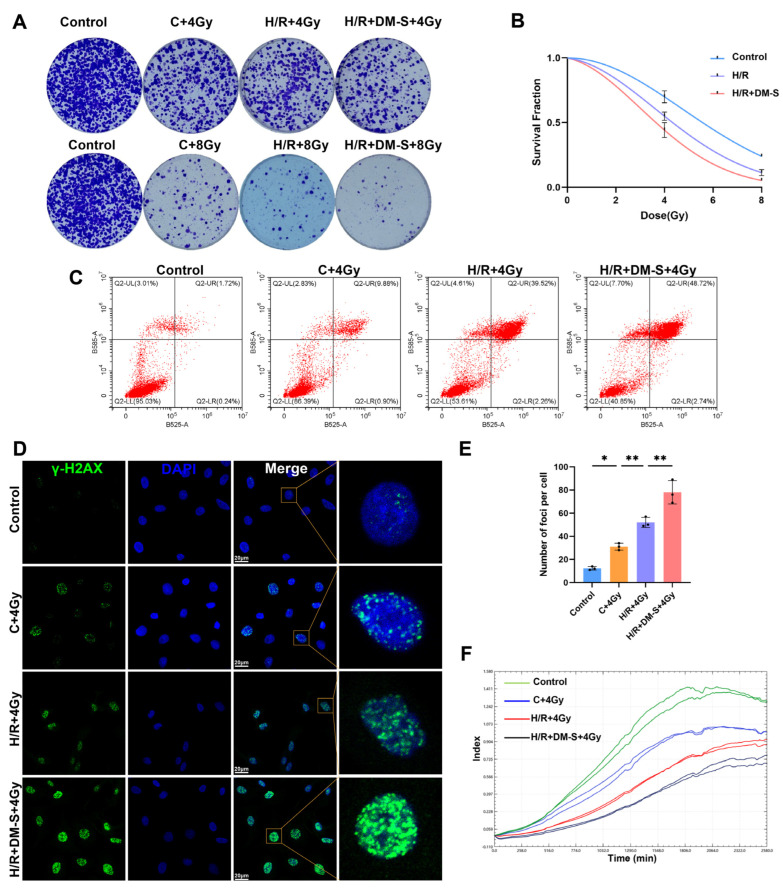
H/R Combined with DM-S Synergistically Enhances Radiosensitivity In Vitro. (**A**) Representative colony formation images of A549 cells (*n* = 3). (**B**) Quantitative analysis of survival fractions from colony formation assays. (**C**) Flow cytometry analysis of apoptosis in A549 cells, measured by annexin V-FITC/PI staining at 24 h post-irradiation (*n* = 3). (**D**,**E**) Immunofluorescence staining for γ-H2AX foci formation in A549 cells (*n* = 5). (**F**) Real-time monitoring of the radiosensitivity index. Data are presented as mean ± SEM. Error bars represent SEM, and statistical significance between groups was analyzed using one-way ANOVA, followed by Tukey’s post hoc test for multiple comparisons. * *p* < 0.05, ** *p* < 0.01.

To investigate the effect of H/R combined with DM-S on tumor cell radiosensitivity in vitro, A549 cells were subjected to irradiation alone, irradiation following H/R, or irradiation following DM-S combined with H/R. Compared to irradiation alone, both H/R + irradiation and DM-S + H/R + irradiation treatments markedly enhanced the inhibitory effect of X-rays on A549 cell proliferation (Figure 5F). Representative colony formation images are shown in Figure 5A, with the corresponding quantitative survival fractions presented in Figure 5B. The results demonstrated that, relative to irradiation alone, H/R pretreatment significantly suppressed the clonogenic capacity of cells after 4 Gy and 8 Gy irradiation, leading to a notable decrease in cell survival fraction. Importantly, the combined intervention of DM-S further augmented the radiation-induced inhibition of colony formation, causing a downward shift in the survival curve. This indicates that DM-S synergizes with H/R to significantly enhance the radiosensitivity of A549 cells to X-rays. To further elucidate the radiosensitizing effect of H/R, apoptosis in A549 cells was assessed by annexin V-FITC/PI staining. At 24 h post-irradiation, the apoptotic rate in the combination treatment group (DM-S + H/R + irradiation) increased to 53.4 ± 1.9% (early apoptosis, 2.6 ± 0.2%; late apoptosis, 50.8 ± 2.1%), in contrast to only 10.3 ± 1.2% (early apoptosis, 0.8 ± 0.2%; late apoptosis, 9.5 ± 1.3%) in the irradiation-alone group (Figure 5C). To more precisely verify the radiobiological effects of H/R and DM-S + H/R, γ-H2AX foci formation was examined in A549 cells. As shown in Figure 5D,E, irradiation following DM-S combined with H/R induced a significant increase in DNA damage, with persistently elevated levels of radiation-induced DNA double-strand breaks.

Importantly, to test whether the observed radiosensitization is cell-line-specific, we validated the key findings in an additional NSCLC cell line, H1299. As shown in Figure S3: (1) Rotenone treatment under normoxia increased ROS, whereas H/R + rotenone reduced ROS, mirroring the A549 results (Figure S3A). (2) DM-S alone did not elevate ROS under control conditions, but significantly increased ROS under H/R (Figure S3B). (3) DM-S + H/R markedly elevated MDA levels, indicating lipid peroxidation (Figure S3C). (4) H/R + 4 Gy irradiation and H/R + DM-S + 4 Gy irradiation significantly increased the apoptotic cell fraction compared to irradiation alone (Figure S3D).

These data confirm that the RET-driven radiosensitization mechanism is not restricted to A549 cells but is reproducible in another NSCLC line with distinct genetic background, supporting the generalizability of our findings.

### 3.6. High Oxygen Combined with DM-S Enhances Radiosensitivity in Hypoxic Tumors In Vivo

**Figure 6:**
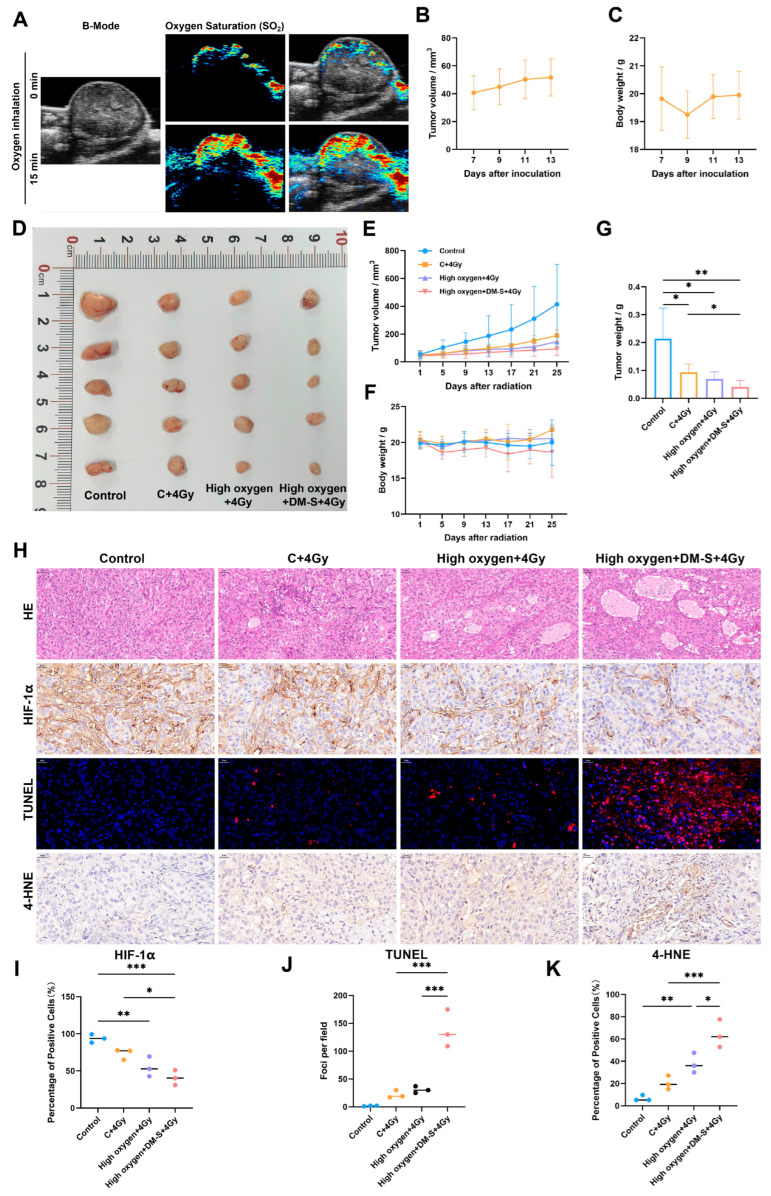
High Oxygen Combined with DM-S Enhances Radiosensitivity in Hypoxic Tumors In Vivo. (**A**) Ultrasound B-mode imaging and oxygen saturation (SO_2_) mapping were used to monitor tumor oxygenation levels before and after a 15-min high oxygen exposure. (**B**) Tumor volume measurements over time post-inoculation (*n* = 5). (**C**) Body weight measurements of mice over the course of the experiment. (**D**) Tumor images showing the effect of treatments: Control, C + 4 Gy, High oxygen + 4 Gy, High oxygen + DM-S + 4 Gy (*n* = 5). (**E**) Tumor growth curves after radiation exposure (*n* = 5). (**F**) Body weight analysis over the duration of the study (*n* = 5). (**G**) Final tumor weights at the end of the observation period (*n* = 5). (**H**) Histological analysis of tumors stained with H&E, showing necrosis and apoptotic morphology, and immunohistochemical staining for HIF-1α, TUNEL, and 4-HNE (*n* = 3) Scale bars: 50 μm (H&E); 25 μm (HIF-1α, TUNEL, and 4-HNE). (**I**–**K**) Quantification of HIF-1α-positive cells, TUNEL-positive cells, and 4-HNE-positive cells across different treatment groups. Data are presented as mean ± SEM. Error bars represent SEM, and statistical significance between groups was analyzed using one-way ANOVA, followed by Tukey’s post hoc test for multiple comparisons. * *p* < 0.05, ** *p* < 0.01, *** *p* < 0.001.

To explore the translational potential of our findings, we evaluated the radiosensitizing effect of succinate combined with high oxygen in a murine xenograft model bearing A549 tumors. Mice received intraperitoneal injection of dimethyl succinate (DM-S, 100 μg/kg) followed by inhalation of 95% O_2_ to recapitulate the metabolic priming and reoxygenation conditions identified in vitro, prior to localized tumor irradiation (4 Gy). Blood oximetry revealed that a 15-min high oxygen exposure significantly increased tumor tissue oxygen saturation, indicating effective amelioration of intratumoral hypoxia (Figure 6A). Tumor growth curves demonstrated that while high oxygen alone before irradiation resulted in a modest, non-significant inhibitory effect, the combination of DM-S and high oxygen before irradiation produced a marked synergistic antitumor response, leading to near-complete growth arrest with no evidence of regrowth during the 25-day observation period (Figure 6D,E). Terminal tumor weights were significantly reduced in the combination group compared to irradiation alone, with a trend toward further reduction relative to high oxygen irradiation (Figure 6G). No significant body weight loss was observed across treatment groups, suggesting minimal systemic toxicity (Figure 6C,F).

Histological examination by H&E staining revealed extensive necrosis and apoptotic morphology exclusively in tumors receiving the combination treatment (Figure 6H). TUNEL staining confirmed a significant increase in apoptosis in the combination group, with TUNEL-positive foci measured at 138.0 ± 33.7, compared to 22.0 ± 7.0 in the IR-alone group and 30.7 ± 6.0 in the high oxygen + IR group (Figure 6H,J). Immunohistochemical analysis further demonstrated that combination treatment exacerbated oxidative lipid damage, as evidenced by a 2.14-fold increase in 4-HNE staining intensity compared to IR alone (Figure 6H,K). Importantly, HIF-1α expression, a marker of tumor hypoxia, was significantly reduced in the combination group (56.5 ± 10.8% reduction vs. control, Figure 6H,I), indicating effective alleviation of intratumoral hypoxia.

Collectively, these in vivo data establish that high oxygen combined with dimethyl succinate acts as a potent radiosensitizer by reactivating mitochondrial reverse electron transfer, driving ROS-mediated oxidative stress, and overcoming hypoxia-induced radioresistance without overt toxicity.

## 4. Discussion

This study unveils a novel radiosensitization mechanism in NSCLC centered on mitochondrial RET. Using an in vitro H/R model to recapitulate the dynamic oxygenation fluctuations characteristic of tumor hypoxic regions, we demonstrate that H/R effectively induces mitochondrial-driven oxidative stress, characterized by a burst of mitochondrial superoxide anions. Mechanistically, this process is not attributable to conventional forward electron transfer but is instead mediated by RET at mitochondrial complex I. Targeted metabolomics identified succinate accumulation during hypoxia as a critical metabolic switch that primes RET, while reoxygenation supplies the requisite high membrane potential and reducing equivalent driving force to execute it. Exogenous supplementation with the membrane-permeable succinate analog DM-S markedly exacerbated H/R-induced mitochondrial oxidative damage, DNA injury, and mitochondrial network fragmentation, and synergistically enhanced the radiosensitivity of A549 cells to X-irradiation. Collectively, these findings provide the first systematic elucidation of the activation conditions, metabolic basis, and therapeutic applicability of RET in radiosensitization within a tumor cell H/R model.

Mitochondrial RET was originally characterized in ischemia–reperfusion (I/R) injury, wherein succinate accumulates during ischemia and is rapidly oxidized by complex II upon reperfusion, driving electrons retrograde from the ubiquinone pool to complex I and triggering a superoxide burst [41,47]. The present study recapitulates this canonical metabolic cascade in a tumor cell H/R model, suggesting that RET is not a pathological phenomenon confined to ischemic organs but may represent a universal stress response to dynamic oxygenation changes within the tumor microenvironment. Notably, while prior investigations have focused on the deleterious consequences of RET in tissue damage, our work reframes RET as a therapeutically actionable vulnerability. By artificially inducing RET to explosively generate ROS in hypoxic tumor regions, we disrupt mitochondrial homeostasis from within, thereby enhancing radiosensitivity. This conceptual pivot repositions RET from a “pathological target to be suppressed” to a “therapeutic lever to be exploited,” representing a fundamental shift in perspective.

Our findings further delineate the dual prerequisites for RET activation: sufficient oxygen availability to support complex II-mediated succinate oxidation, and maintenance of a high ΔΨm to drive reverse electron flow. In the H/R model, hypoxia induced a significant decline in the activities of complexes III, IV, and ATP synthase, while complexes I and II remained functionally intact. This mismatch between upstream and downstream ETC activities, coupled with rapid ΔΨm recovery upon reoxygenation, creates an ideal microenvironment for RET initiation [48]. These observations imply that the metabolic reprogramming experienced by hypoxic tumor regions during reoxygenation may inherently predispose them to RET, whether during intermittent reperfusion in radiotherapy or therapeutic high-oxygen interventions. Consequently, amplifying this signal through exogenous means (e.g., high oxygen combined with succinate precursors) could constitute a highly targeted radiosensitization strategy.

Succinate emerges as the key metabolic driver of RET under H/R conditions. Hypoxia-induced succinate accumulation arises from reverse operation of complex II and inhibition of canonical TCA cycle flux. Supplementation with DM-S significantly potentiated RET-mediated oxidative damage, consistent with prior observations in I/R models [41] and reinforcing succinate’s central role as a “metabolic memory molecule.” Importantly, exogenous succinate elicited a robust ROS burst exclusively under reoxygenation conditions, with negligible effects under normoxia, indicating that its action is strictly contingent upon the oxidative metabolic capacity conferred by restored oxygen supply. This oxygen-dependent requirement confers a degree of tumor selectivity for RET induction: normal tissues, which rarely experience profound hypoxia-reoxygenation fluctuations, are likely less susceptible to succinate plus high-oxygen interventions, thereby mitigating off-target toxicity.

From a radiobiological perspective, this study provides a mechanistic extension of the classical “oxygen fixation hypothesis” [17,24]. Conventional dogma holds that hypoxia-induced radioresistance stems primarily from the absence of molecular oxygen. Under normoxic conditions, the photoelectric effect of X-rays leads to radiolysis of water molecules, generating ROS. Oxygen then reacts with radiation-induced DNA radicals to form peroxyl radicals, a process that ‘fixes’ the damage into permanent, non-repairable clustered lesions (the classic ‘oxygen fixation’ hypothesis). In hypoxic conditions, this fixation is impaired, allowing chemical restitution of DNA radicals and thereby increasing radioresistance. However, our data reveal that hypoxic tumor cells, upon reoxygenation, not only regain radiosensitivity but also exhibit hypersensitivity driven by RET-mediated oxidative stress. DM-S combined with H/R significantly impaired clonogenic survival following 4 Gy and 8 Gy irradiation, elevated apoptosis rates to >50%, and sustained γ-H2AX foci, indicating compromised DNA double-strand break repair. This phenotype is likely linked to RET-induced mtDNA oxidative damage and mitochondrial dysfunction. mtDNA damage has been shown to impair cellular energy metabolism and potentiate radiation-induced nuclear DNA damage [49]. Consistently, longPCR and 8-OHdG assays revealed a significant reduction in mtDNA integrity in the DM-S + H/R group, further supporting this mechanistic axis.

Notably, RET activation concurrently triggered an endogenous antioxidant response in tumor cells, evidenced by upregulation of Nrf2 and downregulation of its repressor Keap1. This feedback adaptation reflects cellular survival mechanisms in response to intense oxidative stress and suggests that sole reliance on RET-induced ROS may not achieve sustained radiosensitization. Combinatorial strategies targeting antioxidant pathways (e.g., Nrf2 inhibitors) could further augment therapeutic efficacy. Moreover, while the feasibility of RET-induced radiosensitization was validated in vitro, in vivo considerations warrant systematic evaluation, including the heterogeneity of tumor hypoxic regions, dynamic blood flow perfusion, and oxygenation status of normal tissues. Future studies employing animal models should explore administration routes, timing, and safety windows for high oxygen combined with succinate analogs or other metabolic activators.

Despite decades of investigation, HBOT has shown limited clinical application as a radiosensitizer for hypoxic tumors. A 2018 systematic review (19 trials, *n* = 2286) demonstrated that HBOT reduces mortality and local recurrence in head and neck cancers only with unconventional fractionation, at the cost of increased severe radiation injury (RR = 2.35), with no clear survival benefit for cervical or bladder cancers [50]. While HBOT is active in mitigating late toxicities (e.g., hemorrhagic cystitis), its radiosensitizing role remains speculative [51], and controversies over dose–response, long-term safety, and pro-angiogenic effects persist [33], underscoring the need for well-designed prospective trials.

In this study, tumor growth inhibition after hyperoxia + DM-S + irradiation did not reach statistical significance versus hyperoxia + irradiation alone (Figure 6G). However, the combination did effectively suppress tumor growth (vs. irradiation alone). Possible reasons include: (i) hyperoxia alone may already saturate oxygen-fixation radiosensitization; (ii) poor DM-S bioavailability in hypoxic niches; (iii) Nrf2-mediated antioxidant feedback. These observations reveal that once tumor oxygenation reaches a certain level, further efficacy gains are limited. Our RET-targeting strategy (succinate + hyperoxia) overcomes this by actively inducing mitochondrial ROS bursts via RET, rather than merely raising oxygen levels. Although not significant in vivo, robust synergy in vitro and in H1299 cells validates the mechanism. Future optimization (e.g., improved DM-S delivery, Nrf2 inhibition, more hypoxic models) may achieve true radiosensitization beyond hyperoxia, providing a new basis to tackle the translational hurdles of HBOT.

It is also important to acknowledge several experimental limitations in our in vivo study. Owing to animal availability constraints and strict adherence to the 3R principle (Replacement, Reduction, Refinement), we were unable to include control groups receiving DM-S alone, high oxygen alone (without radiation), or DM-S + radiation (without high oxygen). Consequently, the independent contribution of each component-DM-S, high oxygen, and radiation-to the observed tumor growth inhibition cannot be fully delineated. The in vivo findings should therefore be considered preliminary, and future studies incorporating these missing control groups are warranted to confirm the specific role of DM-S in hyperoxia-enhanced radiosensitization. Moreover, the sample size (*n* = 5 per group) was modest in the absence of an a priori power analysis. These in vivo findings should therefore be considered preliminary and warrant confirmation in future studies with larger cohorts and complete control groups. Nevertheless, the differences between key treatment groups (e.g., high oxygen + DM-S + 4 Gy vs. 4 Gy) reached statistical significance, and the effect sizes were consistent with our mechanistic predictions.

In conclusion, this study systematically elucidates the mechanisms governing RET activation in tumor cells under H/R conditions and its metabolic regulation, proposing a novel strategy: artificially activating RET to induce a mitochondrial oxidative storm, thereby enhancing radiosensitivity. This approach integrates mitochondrial metabolic reprogramming with radiotherapy, offering new theoretical foundations and interventional targets for the treatment of hypoxic solid tumors such as NSCLC. Future research will focus on optimizing RET activation strategies and evaluating their translational potential when integrated into clinical radiotherapy protocols.

## Figures and Tables

**Table 1:** COX II primer sequences used in this study.

Gene	Gene ID	Primer Direction	Sequence (5′→3′)
COX II	4513 (accession number NC_012920.1)	Forward primer	CACTCCACGGAAGCAATA
		Reverse primer	AAATGAATGAGCCTACAGA

**Table 2:** Primary antibody source and dilution ratio.

Antibody Name	Brand	Item Number	Dilution Ratio
NDUFA9	Selleck	A24H16	1:1000
SDHC	Selleck	E11N4	1:5000
UQCRC2	Selleck	K17L12	1:2000
MTCO2	Selleck	A18M9	1:5000
ATP5A	Selleck	H18N20	1:1000
NRF2	Abmart	T55136F	1:1000
KEAP1	Proteintech	10503-2-AP	1:3000

**Table 3:** Primer sequences used in this study.

Gene	Gene ID	Primer Direction	Sequence (5′→3′)
mtND1	4535 (accession number NC_012920.1)	Forward primer	ATACCCATGGCCAACCTC
		Reverse primer	TAGGTTTGAGGGGGAATGC
mtDNA	NCBI Reference Sequence: NC_012920.1	Forward primer	TGAGGCCAAATATCATTCTGAGGGGC
		Reverse primer	TTTCATCATGCGGAGATGTTGGATGG

**Table 4:** Long PCR reaction system table.

Component	50 μL Reaction System	Final Concentration
2× GoTaq Long PCR MasterMix	25 μL	1×
Upstream Primer	20 pmol	0.4 μM
Downstream Primer	20 pmol	0.4 μM
Template DNA	<0.5 μg	<0.5 μg
Nuclease-free water	Up to 50 μL	

**Table 5:** Long PCR reaction condition table.

Step	Temperature (°C)	Time
Initial Denaturation	95	2 min
Denaturation	93	20 s
Annealing	53	25 s
Extension	65	17 min
Final Extension	72	10 min
Hold	4	∞

A total of 30 cycles was set. The first 15 cycles were performed under normal conditions (as listed above). For the remaining 15 cycles, an additional 15 s of extension time was added per cycle.

## Data Availability

The original contributions presented in this study are included in the article/Supplementary Materials. Further inquiries can be directed to the corresponding authors.

## Supplementary Materials

The following supporting information can be downloaded at: https://www.mdpi.com/article/10.3390/antiox15060697/s1, Figure S1: Enzymatic activity of mitochondrial complex V (ATP synthase) in A549 cells under different conditions; Figure S2: Mitochondrial oxygen consumption was measured using a Clark-type oxygen electrode and the respiratory control ratio (RCR) was calculated as state 3 divided by state 4; Figure S3: Validation of RET-driven radiosensitization in H1299 NSCLC cells.

## Abbreviations

The following abbreviations are used in this manuscript:

NSCLC	Non-small Cell Lung Cancer
RET	Reverse Electron Transfer
H/R	Hypoxia/Reoxygenation
ROS	Reactive Oxygen Species
DM-S	Dimethyl Succinate
HIFs	Hypoxia-inducible Factors
mPTP	Mitochondrial Permeability Transition Pore
OXPHOS	Oxidative Phosphorylation
CoQ	Coenzyme Q
COX II	Cytochrome C Oxidase Subunit II
DCFH-DA	2′,7′-Dichlorodihydrofluorescein Diacetate
DHE	Dihydroethidium
MFI	Mean Fluorescence Intensity
ΔΨm	Mitochondrial Membrane Potential
MDA	Malondialdehyde
ETC	Electron Transport Chain
I/R	Ischemia–reperfusion
DMM	dimethyl malonate
