# Supplementary material for: Hypoxia/Reoxygenation-Induced Mitochondrial Reverse Electron Transfer: A Targetable Mechanism to Enhance Radiosensitivity in Non-Small Cell Lung Cancer
Source: Antioxidants (Basel). 2026 May 31;15(6):697. doi: 10.3390/antiox15060697 (PMC13295712; doi:10.3390/antiox15060697)
Supplement: Supplementary file 1 [file antioxidants-15-00697-s001.zip › antioxidants-4265945-supplementary.pdf]

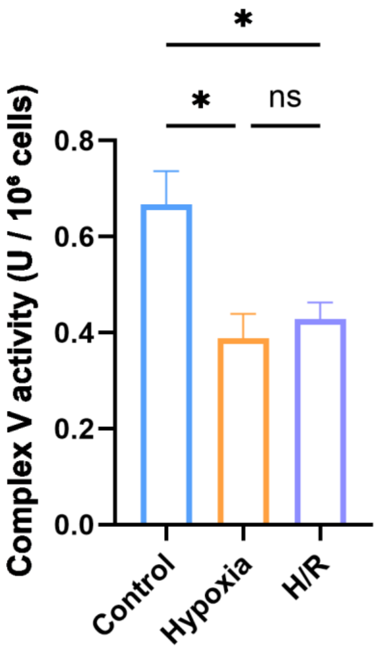

Figure S1. Enzymatic activity of mitochondrial complex V (ATP synthase) in A549 cells under different conditions. The enzymatic activity of mitochondrial complex V (ATP synthase) was determined by a spectrophotometric method based on an ATP hydrolysis-coupled assay (n=3). All the data are presented as mean ± SEM from at least three independent experiments, and error bars represent SEM. Statistical significance between groups was analyzed using one-way ANOVA, followed by Tukey's post-hoc test for multiple comparisons. *p<0.05, "ns" represents no statistical difference.

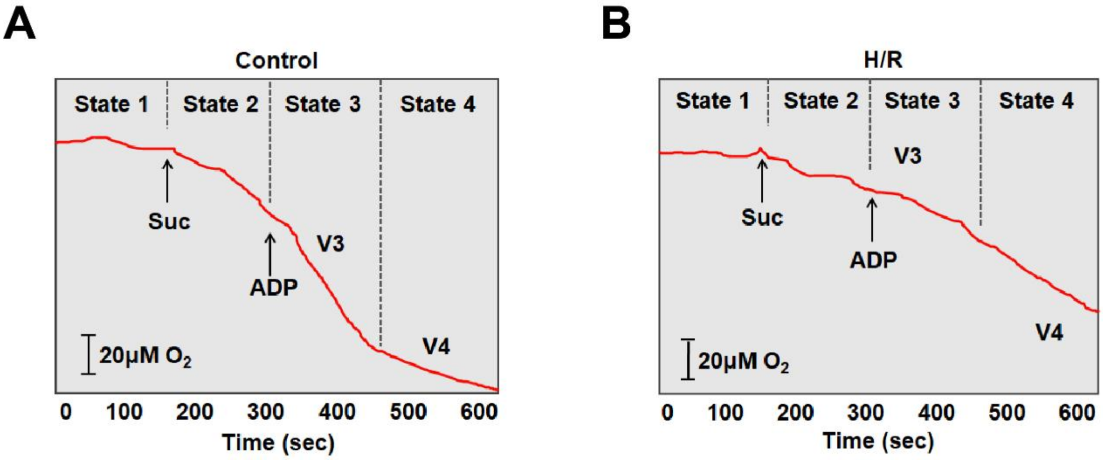

V3	V4	RCR
360 nM/s	110 nM/s	3.27

V3	V4	RCR
135 nM/s	131 nM/s	1.03

Figure S2. Mitochondrial oxygen consumption was measured using a Clark-type oxygen electrode and the respiratory control ratio (RCR) was calculated as state 3 divided by state 4.

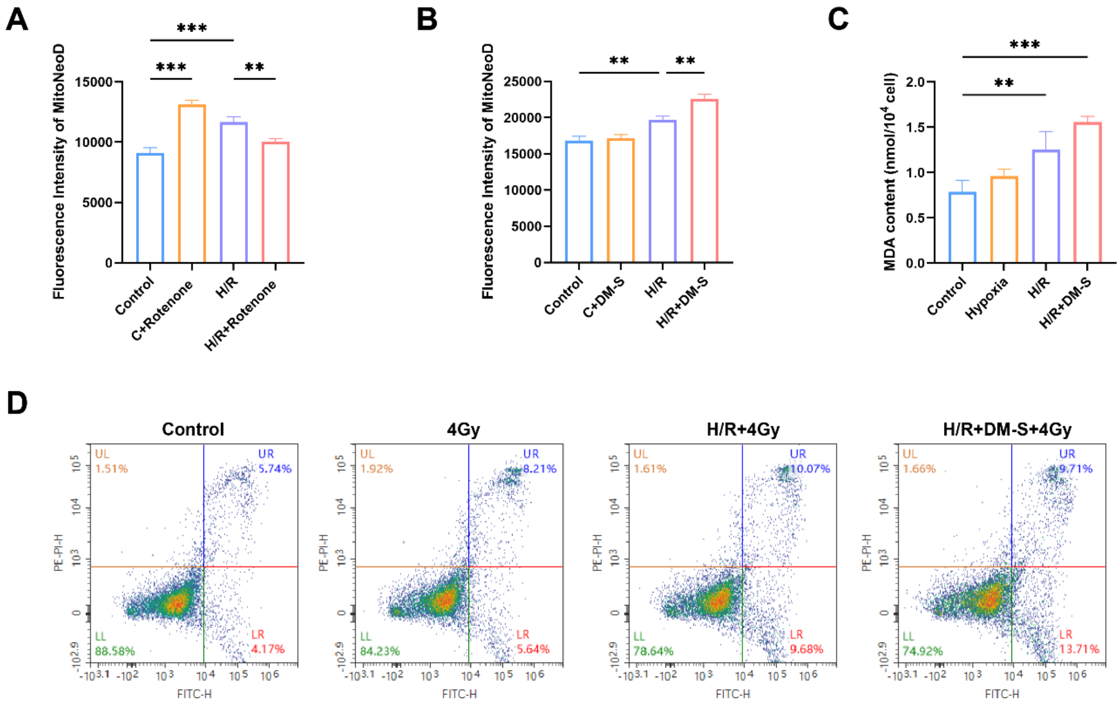

Figure S3. Validation of RET-driven radiosensitization in H1299 NSCLC cells. (A-B) mitochondrial ROS levels were assessed by measuring MitoNeoD fluorescence intensity (n=3). (C) MDA levels under different treatment conditions. MDA is a marker of lipid peroxidation(n=3). (D) Flow cytometry analysis of apoptosis in H1299 cells, measured by annexin V-FITC/PI staining at 24 hours post-irradiation (n=3). All the data are presented as mean ± SEM from at least three independent experiments, and error bars represent SEM. Statistical significance between groups was analyzed using one-way ANOVA, followed by Tukey's post-hoc test for multiple comparisons. $^{**}p<0.01$, $^{***}p<0.001$.

Mrthods

Mitochondrial Respiration Assay Using a Clark Oxygen Electrode:

Mitochondrial oxygen consumption was measured using a Clark-type oxygen electrode (Hansatech Instruments, Norfolk, UK) at 37 °C in mitochondrial respiration buffer (125 mM KCl, 5 mM K_2HPO_4, 20 mM HEPES, 2.5 mM EGTA, and 1 mM $MgCl_2 \cdot 6H_2O$; pH 7.2). Succinate (10 mM) was used as the substrate, and the mitochondrial protein concentration was adjusted to 500 µg/mL. ADP-stimulated oxygen consumption (state 3 respiration) was measured in the presence of 1 mM ADP, and state 4 respiration was recorded following ADP depletion. The respiratory control ratio (RCR) was calculated as state 3 divided by state 4 to assess mitochondrial coupling.
